# LIX1-like protein drives hepatic stellate cell activation to promote liver fibrosis by regulation of chemokine mRNA stability

**DOI:** 10.1038/s41392-021-00665-6

**Published:** 2021-09-01

**Authors:** Xiaoyun Zhu, Yanqiu Zhang, Yucheng Zhao, Dejuan Xiang, Jie Zou, Ourania Andrisani, Hao Zhang, Lingyi Kong

**Affiliations:** 1 Jiangsu Key Laboratory of Bioactive Natural Product Research and State Key Laboratory of Natural Medicines, School of Traditional Chinese Pharmacy, China Pharmaceutical University, Nanjing, China; 2 Department of Basic Medical Sciences and Purdue Center for Cancer Research, Purdue University, West Lafayette, IN USA

**Keywords:** Disease model, Gastrointestinal diseases

**Dear Editor**,

Hepatic stellate cells (HSCs) play a key role in the fibrotic response, thus inactivating activated HSC could be a potential therapy for fibrosis.^1,2^ CCL20 expressed by HSCs and macrophages, may serve as a mediator of inflammation and fibrosis.^3^ LIX1L is a putative RNA-binding protein (RBP) that may play an important role in post-transcriptional gene regulation.^4^ However, the biological function of LIX1L in liver fibrosis remains unclear, we therefore aimed to characterize its functions in HSC activation and liver fibrosis.

*LIX1L* expression was significantly upregulated in human cirrhotic liver (GSE25097), tumor tissue, and patients with fibrotic livers, and was positively correlated with liver fibrosis stage, level of inflammatory and α-SMA expression in cirrhotic liver (Supplementary Fig. s1a, b and Fig. 1a). Similarly, LIX1L expression was markedly increased in CCl_4_ or BDL induced mouse model of liver fibrosis (Fig. 1b).

**Fig. 1:**
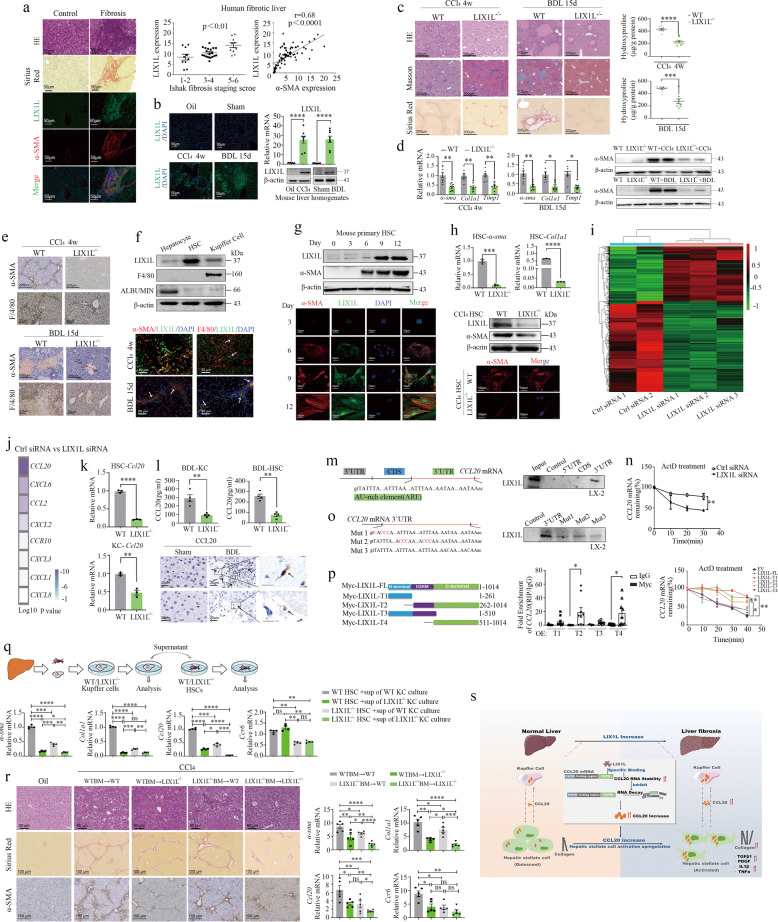
LIX1L drives HSC activation to promote liver fibrosis by regulating chemokine mRNA stability. **a** Sirius Red staining and double immunofluorescence staining for LIX1L (green), α-SMA (red) in normal and fibrotic human livers (30 normal and 39 fibrotic liver tissue spots). The positive staining areas were measured by Image J software. Scale bar: 50 μm. Correlation analyses of LIX1L expression with Ishak fibrosis staging scores of Sirius Red-stained sections (left). The positive correlation of LIX1L with α-SMA in patients with fibrosis from tissue arrays (right). **b** Representative immunofluorescence staining and western blot analysis of LIX1L in mouse livers from WT mice with or without CCl_4_ or BDL treatment (*n* = 8 per group). **c** Representative images of H&E, Sirius Red, Masson’s trichrome staining from liver tissues of WT littermates and *Lix1l*^*−/−*^ mice. Scale bar: 200 μm (left). Liver fibrosis was evaluated by hydroxyproline content (right) (*n* = 8 per group). **d** Hepatic mRNAs of fibrogenic genes were measured by RT-qPCR assays in WT and *Lix1l*^*−/−*^ mice treated with CCl_4_ or BDL (left). Immunoblotting analysis of α-SMA expression in the mouse liver (left) (*n* = 8 per group). **e** Representative images of IHC staining for α-SMA, F4/80 from liver tissues of WT littermates and *Lix1l*^*−/−*^ mice treated with CCl_4_ or BDL. Scale bar: 200 μm, 20 μm (*n* = 8 per group). **f** Immunoblotting analysis of LIX1L expression in isolated and cultured Hepatocytes, KCs, and 9-day cultured HSCs, from WT mice without treatment (up). Double immunofluorescence staining for LIX1L (green), F4/80(red), and α-SMA (red) in the livers from BDL and CCl_4_ mice. Nuclei were counter-stained with DAPI (blue). Scale bar: 50 μm (bottom). **g** Western blot analysis of LIX1L and α-SMA expression in cultured primary HSCs from WT mice without treatment at the indicated times (up). Double immunofluorescence staining for LIX1L (green) and α-SMA (red) in cultured primary HSCs from WT mice. Scale bar: 50 μm (bottom). **h** Fibrogenic gene expression in cultured HSCs (9 days) isolated from WT and *Lix1l*^*−/−*^ mice without treatment (up). Immunoblotting assays of LIX1L and α-SMA in cultured HSCs from WT and *Lix1l*^*−/−*^ mice with CCl_4_ treatment (middle). Immunofluorescence staining for α-SMA (red) on HSCs from WT and *Lix1l*^*−/−*^ mice treated with CCl_4_. Nuclei were counter-stained with DAPI (blue). Scale bar: 50 μm (bottom). **i** Heatmaps of genes involved in fibrosis and chemokine signaling pathway from RNA-seq analysis. LX-2 cells were transfected with control or LIX1L siRNAs for 48 h and the gene expression change was measured by RNA-seq. **j** Heatmap showing the differentially expressed genes related to the chemokine signaling pathway. **k** Primary HSCs and KCs were isolated from WT and *Lix1l*^*−/−*^ mice without treatment and cultured for 6 days. *CCL20* mRNA was measured by RT-qPCR assays. **l** CCL20 secretion was measured in the supernatant of cultured primary HSCs (6 days) and KCs (3 days) isolated from WT and *Lix1l*^*−/−*^ mice with BDL treatment (up). IHC staining of CCL20 in livers from WT or *Lix1l*^*−/−*^ mice with BDL treatment (*n* = 8 per group). Scale bar: 20 μm (bottom). **m** Top, schematic of human *CCL20* mRNA with AREs. Bottom, RNA pull-down followed by western blot. Cell lysates of LX-2 cells were incubated with biotin-labeled 5′ UTR, CDS, 3′ UTR of *CCL20* mRNA, or a negative control transcript. After pull-down, the recruitment of LIX1L to *CCL20* 3′ UTR was examined by western blot. **n** LX-2 cells transfected with control or LIX1L siRNA were treated with actinomycin D (1 µg/mL). RNA was extracted at indicated time points (0, 10, 20, 30 min) and *CCL20* mRNA was analyzed by RT-qPCR. **o** Top, schematic of *CCL20* 3′ UTR with mutated AREs. Bottom, Western blot of LIX1L after RNA pull-down. **p** Left, different truncated mutants of LIX1L. Middle, RIP assays to determine the interaction between *CCL20* mRNA (full length) and LIX1L mutants employing lysates from LX-2 cells transfected with empty vector (EV) or Myc-tagged LIX1L mutant. Right, LX-2 cells transfected with LIX1L mutant were treated with actinomycin D (1 µg/mL). RNA was extracted at different time points (0, 10, 20, 30, 40 min) and *CCL20* mRNA was analyzed by RT-qPCR. **q** Primary HSCs from WT or *Lix1l*^*−/−*^ mice without treatment were incubated with the supernatant from WT or *Lix1l*^*−/−*^ KCs (top). Expression of the fibrogenic genes, *Ccl20* and *Ccr6* were analyzed by RT-qPCR. **r** Chimeric mice were generated by transplanting WT or *Lix1l*^*−/−*^ BM into irradiated and clodronate-treated WT or *Lix1l*^*−/−*^ mice. Liver fibrosis was induced by 12 injections of CCl_4_ for 4 weeks (*n* = 5 per group). Representative histology of H&E, Sirius Red, and IHC staining are shown. Scale bar: 50 μm (left). Hepatic mRNA levels of fibrogenic genes and *Ccl20*, *Ccr6* were analyzed by RT-qPCR (right). **s** A schematic diagram illustration of LIX1L promoting liver fibrosis through regulating CCL20 expression. Data are presented as means ± SEM. *P < 0.05; **P < 0.01; ***P < 0.001; ****P < 0.0001

Next, *Lix1l* knockout (*Lix1l*^*−/−*^) mice were generated and subjected to CCl_4_ and BDL treatment. H&E staining, Masson′s trichrome, and Sirius red staining, together with biochemical collagen quantification results showed decreased collagen deposition and pseudo lobular nodule formation in *Lix1l*^*−/−*^ mice. In addition, serum ALT and AST, proinflammation genes, and α-SMA protein expression were dramatically reduced in *Lix1l*^*−/−*^ mice compared to WT mice (Fig. 1c, d and Supplementary Fig. s2a, b).

HSC and KC activation play important role in the process of liver fibrosis. The fibrogenic activity of HSC and hepatic macrophage infiltration as indicated by α-SMA and F4/80 expression was reduced in *Lix1l*^*−/−*^ mice (Fig. 1e). To determine the cellular distribution of LIX1L, we examined LIX1L expression in cultured primary HSCs, KCs, and hepatocytes. Primary cells isolated from healthy, CCl_4_ and BDL treated mice showed that LIX1L was more abundant in HSCs and KCs than in hepatocytes. Dual immunofluorescence staining of liver tissues displayed a co-localization of LIX1L with α-SMA-positive HSCs and F4/80-positive macrophages (Fig. 1f and Supplementary Fig. s3a, b). To confirm these results, we employed adeno-associated viral (AAV)-LIX1L-Flag to express LIX1L in hepatocytes of LIX1L knockout mice. Of note, LIX1L overexpression in hepatocytes failed to reverse the beneficial effect of LIX1L knockout on fibrosis (Supplementary Fig. s3c–f), indicating that LIX1L in HSC and KCs predominantly regulating liver fibrosis.

HSC activation is key event in the development of liver fibrosis. We tested whether increased LIX1L regulated HSC activation and found that LIX1L levels gradually increased in primary HSCs during culture activation, with induction of α-SMA (Fig. 1g and Supplementary Fig. s4a). LIX1L deletion inhibited fibrogenic markers expression in primary HSCs and LX-2 cells and inhibited HSC activation in CCl_4_ challenged mice, while LIX1L overexpression further enhanced α-SMA and fibrogenic markers expression in LX-2 cells (Fig. 1h and Supplementary Fig. s4b–f). Together, these results suggest that LIX1L promotes liver fibrosis involving in regulation of HSC activation.

To identify the mechanism(s) underlying LIX1L-induced HSC activation, we compared gene expression profiles of control and LIX1L knockdown LX-2 cells (Supplementary Fig. s6a). The results revealed that LIX1L knockdown reduced the expression of a wide spectrum of genes known to play critical roles in HSC activation and liver fibrosis (Fig. 1i). KEGG pathway analysis revealed that genes repressed by LIX1L knockdown were most enriched in the chemokine signaling pathway. Meanwhile, the expression of CCL20 and CCL2 was significantly reduced, among which CCL20 was reduced the most (Fig. 1j and Supplementary Fig. s6b, c). *Ccl20* mRNA and CCL20 expression by Elisa assay were significantly reduced in LIX1L-deficient HSCs and KCs (Fig. 1k and Supplementary Fig. s6g, h), as well as the supernatant of cultured primary HSCs and KCs isolated from mice with BDL treatment (Fig. 1l), suggesting that LIX1L is required for CCL20 production in these cell types. Immunohistochemical analyses revealed that CCL20 staining was mostly in non-parenchymal cells, and reduced in *Lix1l*^*−/−*^ mice treated with CCl_4_ or BDL (Supplementary Fig. s6 i, j). In addition, CCL20 knockdown significantly reduced fibrotic gene expression in LX2 cells (Supplementary Fig. s6k). CCL20 could activate HSCs and reverse the inhibitory effect of LIX1L deletion on HSC activation (Supplementary Fig. s6l). Moreover, exogenous CCL20 could upregulate the expression of *Lix1l* in HSCs and KCs (Supplementary Fig. s6m). Given that endogenous CCL20 expression was decreased in *Lix1l*^−/−^ KCs compared to WT KCs. Therefore, in order to determine whether endogenous CCL20 affects the expression of LIX1L, we detected the expression of LIX1L in primary HSCs co-cultured with *Lix1l*^−/−^ KCs and WT KCs. We found that endogenous CCL20 could upregulate the expression of LIX1L (Supplementary Fig. s6n). Together, these data further suggest that LIX1L promotes HSC activation through the regulation of CCL20 signaling pathway.

RNA immunoprecipitation assay indicated that *CCL20* mRNA was enriched by LIX1L (Supplementary Fig. s7a). RNA pulldown assay showed that 3′ UTR region of *CCL20* mRNA recruited LIX1L protein (Fig. 1m), suggesting that LIX1L may directly influence the expression of CCL20 mRNA at the post-transcriptional level. Indeed, Gene Set Enrichment Analysis (GSEA) identified LIX1L knockdown regulating RNA stability (Supplementary Fig. s7b), and LIX1L knockdown facilitated *CCL20* mRNA degradation (Fig. 1n). Thus, LIX1L knockdown promoted CCL20 mRNA decay. The mRNAs of many chemokines and cytokines are targeted for rapid degradation through AU-rich elements (AREs) located in their 3′ UTR.^5^ We tested whether AREs are required for LIX1L binding, and found that these AREs in 3′ UTR of *CCL20* mRNA are essential for LIX1L binding (Fig. 1o). Next, we found that *CCL20* mRNA was only enriched with LIX1L mutants that contained C-terminal region, LIX1L mutants which could bind *CCL20* mRNA stabilized *CCL20* mRNA and promoted fibrogenic genes expression, while mutants lacking RNA-binding ability lost this promoting role (Fig. 1p and Supplementary Fig. s7d). Together, these data suggest that LIX1L binding to AREs in 3′ UTR of *CCL20* mRNA leads to its stabilization.

Given that LIX1L is important for HSC activation and liver fibrosis, and LIX1L is highly expressed in activated HSCs and KCs, we next investigated the contribution of KCs to LIX1L-induced fibrogenesis. The supernatant from WT or *Lix1l*^*−/−*^ KCs was added to a primary culture of WT and *Lix1l*^*−/−*^ HSCs for 3 days. Our results demonstrated significant downregulation of profibrogenic markers, *Ccl20* and *Ccr6*, in *Lix1l*^*−/−*^ HSCs exposed to WT or *Lix1l*^*−/−*^ KCs supernatant (Fig. 1q). Of note, the expression of CCL20 by Elisa assay was similar to the aforementioned results (Supplementary Fig. s8a). We generated LIX1L chimeric mice followed by CCl_4_ treatment to further investigate which cell types are critical in LIX1L-mediated liver fibrosis (Supplementary Fig. s8b, c). Strikingly, these results further verified that LIX1L in liver resident cells contributes to liver fibrosis. Reduced fibrosis correlated with lower expression of fibrogenic genes, *Ccl20* and *Ccr6*, lower α-sma expression, as well as serum ALT and AST activities (Fig. 1r and Supplementary Fig. s8d–h). Taken together, these results suggest that LIX1L signaling in liver resident HSCs and KCs is required for the pathogenesis of liver fibrosis, whereas LIX1L in BM-derived cells plays a lesser role in liver fibrosis.

In summary, our study identified LIX1L as a novel regulator of liver fibrosis through activation of HSCs during chronic liver injury. The interaction of LIX1L with CCL20 mRNA acts as key molecular event that connects CCL20-CCR6 axis, KCs-HSCs communication, and activation of HSCs. Hence, LIX1L inhibition may be a promising strategy for the prevention of hepatic fibrosis.

## Supplementary information

Supplemental information PDF-clean

## Data Availability

The datasets produced in this study are available in the following databases: RNA‐seq data for cortical organoids: Gene Expression Omnibus GSE133121 (GEO: http://www.ncbi.nlm.nih.gov/geo/).
