# Supplementary material for: LIX1-like protein drives hepatic stellate cell activation to promote liver fibrosis by regulation of chemokine mRNA stability
Source: Signal Transduct Target Ther. 2021 Sep 1;6:319. doi: 10.1038/s41392-021-00665-6 (PMC8408256; doi:10.1038/s41392-021-00665-6)
Supplement: Supplementary file 1 — Supplemental information PDF-clean [file 41392_2021_665_MOESM1_ESM.pdf]

LIX1-like Protein drives hepatic stellate cell activation to promote liver fibrosis by regulation of chemokine mRNA stability

Xiaoyun Zhu[1], Yanqiu Zhang[1], Yucheng Zhao[1], Dejuan Xiang[1], Jie Zou[1], Ourania Andrisani[2], Hao Zhang[1]*, Lingyi Kong[1]*

1 Jiangsu Key Laboratory of Bioactive Natural Product Research and State Key Laboratory of Natural Medicines, School of Traditional Chinese Pharmacy, China Pharmaceutical University, Nanjing 210009, China

2 Department of Basic Medical Sciences and Purdue Center for Cancer Research, Purdue University, West Lafayette IN 47907, USA

*Correspondence authors:

Hao Zhang, State Key Laboratory of Natural Medicines and Jiangsu Key Laboratory of Bioactive Natural Product Research, School of Traditional Chinese Pharmacy, China Pharmaceutical University, 24 Tong Jia Xiang, Nanjing 210009, China. E-mail: zhanghao@cpu.edu.cn;

Lingyi Kong, State Key Laboratory of Natural Medicines and Jiangsu Key Laboratory of Bioactive Natural Product Research, School of Traditional Chinese Pharmacy, China Pharmaceutical University, 24 Tong Jia Xiang, Nanjing 210009, China. E-mail: cpu_lykong@126.com;

This PDF file includes:

Extended Results and Discussion

Materials and Methods

Tables S1 to S2

Figures. S1 to S8

Extended Results and Discussion

In the current study, we found that enhanced LIX1L expression correlated with HSC activation and liver fibrosis severity in human liver biopsies. Employing *Lix1l*$^{-/-}$ mice, we verified that genetic inactivation of *Lix1l* attenuated liver fibrosis. Moreover, our results showed that LIX1L binds to *CCL20* mRNA and prevents its decay. Thus, our mechanistic studies, together with clinical data, strongly suggest that LIX1L may serve as an attractive target for antifibrotic therapy.

Reports concerning the expression and function of LIX1L in liver physiology and pathology are scarce. Although LIX1L has been reported to modulate fat signaling to regulate fat levels[1], the role of LIX1L in liver fibrosis has yet to be defined. Here, we demonstrate a strong up-regulation of LIX1L in activated HSCs and fibrotic liver, suggesting that increased LIX1L expression is positively correlated with liver disease progression. We gain insight into the role of LIX1L in hepatic fibrosis, we first generated global *Lix1l* knockout (*Lix1l*$^{-/-}$) mice. No apparent phenotypic changes were seen in *Lix1l*$^{-/-}$ mice (Supplementary Fig. s1c-f). *Lix1l* knockout attenuated CCl_4 and BDL induced chronic liver injury and fibrosis in mice. However, LIX1L deficiency did not affect proliferation and apoptosis of hepatocytes in both models of chronic liver injury (Supplementary Fig. s2c). In addition, *Lix1l* knockout showed no protect role in acute hepatocyte damage (Supplementary Fig. s2d-g), indicating that LIX1L may be involved in HSC activation. Therefore, results of LIX1L deficiency in acute and chronic liver injury mouse model indicating that instead of affecting acute hepatocyte damage, LIX1L participated in HSC activation followed by chronic liver injury induced hepatic

inflammation. Indeed, LIX1L up-regulation was observed in HSCs and KCs isolated from mice treated with CCl_4 and BDL, but not in primary hepatocytes. Together, these findings support the conclusion that LIX1L knockout prevents progression of liver fibrosis if applied onset of chronic liver injury. LIX1L may be a potential therapeutic target for liver fibrosis.

One critical question is how LIX1L promotes liver fibrosis. HSCs activation is a central driver of fibrosis in experimental models and human liver injury[2] . LIX1L deficiency impairs HSC activation *in vitro* and *in vivo*. However, LIX1L knockdown did not affect apoptosis or proliferation of LX-2 cells (Supplementary Fig. s4 g, h). TGFβ1 and PDGF-triggered alteration of α-SMA and COL1A1 expression and HSCs activation were partially compromised by LIX1L silencing (Supplementary Fig. s5). Further pathway analysis showed that LIX1L knockdown highly affected the chemokine signaling pathway, and *CCL20* is the most decreased chemokine. CCL20 could activate HSCs and is involved in chronic liver inflammation and fibrosis [3,4]. Previous literature reported that HSCs and macrophages could express CCL20, and CCL20 in turn further activate HSCs and exert proinflammatory and profibrogenic effects. Interesting, IHC results showed that CCL20 was expressed mostly in non-parenchymal cells, and LIX1L deficiency down-regulated CCL20 expression. In, addition, given that CCL20 was mainly secreted by HSCs and macrophages, we confirmed that LIX1L knockdown decreased, while LIX1L overexpression enhanced CCL20 expression (Supplementary Fig. s6d, e). The expression of CCL20 mRNA and fibrogenic genes were significantly increased in HSCs exposed to EV or Myc-LIX1L

KCs supernatant (Supplementary Fig. s6f). The CCL20 receptor, CCR6 expression showed similar results in aforementioned studies (Supplementary Fig. s6 o-q).

Mechanistically, LIX1L stabilized *CCL20* mRNA via binding to AREs in the 3' UTR of *CCL20* mRNA. LIX1L knockdown promoted *CCL20* mRNA decay. Notably, only one ARE is present in 3' UTR of *CCL2* mRNA according to ARE database, this may account for why loss of LIX1L showed a less significant effect on CCL2 expression as compared to CCL20 (Supplementary Fig. s7c). Compared to *CCL20* and *CCL2*, we noticed that LIX1L has less significant effect on other chemokines (*CXCL2*, *CXCL3* and *CXCL6*), suggesting that other ARE-binding proteins may regulate mRNA stability of these factors. Specifically, the ARE-binding protein Hu antigen R functions as a stabilizing factor[5], MyD88-mediated stabilization of chemokine mRNA is dependent on the presence of 3' UTR ARE[6]. Herein, we observed the LIX1L-mediated mRNA stabilization of *CCL20* is a key molecular event that connects chemokine expression and activation of HSCs.

In summary, activation of HSCs under pathological conditions results in a marked induction of LIX1L. Furthermore, LIX1L enhanced the stability and expression of CCL20 mRNA. CCL20 activated HSCs and further increased LIX1L expression. As for the mechanisms underlying the up-regulation of LIX1L in activated HSCs and fibrotic liver, we speculated that classical HSC-activation cytokines including TGFβ and PDGF, chemokines and signalings may form a positive feedback with LIX1L. However, the detailed mechanisms of LIX1L increase in the process of liver fibrosis need further study.

During the development of liver fibrosis, HSCs, KCs, hepatocytes, LSECs and other cells are involved in the regulation of the occurrence and development of fibrosis. To fully elucidate the role of LIX1L in liver fibrosis is will be necessary to characterize and identify its cell-type-specific functions. HSCs are the source and target of chemokines[7-9], and the interaction of HSCs with proinflammatory cells such as KC is a crucial event in HSC activation and fibrosis, where chemokines and their receptors are likely to serve as important contributors to this interaction[10]. Thus, cell-type specific expression of LIX1L may assist in determining the outcome of liver disease. LIX1L is highly expressed in activated HSCs and KCs. CCL20, mainly expressed by macrophages and HSCs, exerts proinflammatory and profibrogenic effects in liver pathological processes [11,12]. Our present data provide compelling evidence that LIX1L regulates CCL20 expression in KCs and HSCs. Furthermore, we identified a paracrine effect of LIX1L-mediated CCL20 secretion produced by KCs on HSC activation. In this study, in the *in vitro* primary co-culture experiment, we found that the expression of α-SMA, Collα1 and CCL20 in *Lix1l*$^{-/-}$ HSCs with *Lix1l*$^{-/-}$ KCs supernatant was decreased than WT HSCs with *Lix1l*$^{-/-}$ KCs supernatant, as well as than *Lix1l*$^{-/-}$ HSCs with WT KCs supernatant.

Cellular heterogeneity in the liver is partly explained by the origin of macrophages. [13]. To identify the most relevant target cell of LIX1L-mediated profibrotic effects, WT or *Lix1l*$^{-/-}$ mice were reconstituted with BM from WT or *Lix1l*$^{-/-}$ donors. We removed the macrophages from the recipient mice, and the BMDM from the donor mice was partially differentiated into Kupffer cells after being transplanted into the recipient mice.

It was consistent with in vitro experiment, the expression of fibrogenic genes in *Lix1l*$^{-/-}$ BM→*Lix1l*$^{-/-}$ was lower than *Lix1l*$^{-/-}$ BM→ *Lix1l*$^{-/-}$, as well as than *Lix1l*$^{-/-}$ BM→WT, demonstrating that LIX1L signaling in liver resident cells strongly contributes to the pathogenesis of liver fibrosis. The results of *Lix1l* chimeric mice indicated that LIX1L in liver resident cells is a major contributor to liver fibrosis. Since KCs are heterogenic cell population from the BM and liver resident, we conclude that ablation of LIX1L signaling in liver resident HSCs and KCs was critical for inhibition of liver fibrosis. Therefore, targeting LIX1L in liver resident cells may serve as a target for the treatment of liver fibrosis in the future.

Materials and Methods

Animal maintenance and treatment

The *Lix1l*-knockout (KO) mice were generated with crispr-cas9 system (Bioray Laboratories Inc., Shanghai, China), the sgRNA were designed to target LIX1L domain. *Lix1l*$^{-/-}$ with gRNA1: 5′-GAATGTGGTGGAGGCCCTTCAGG-3′; gRNA2: 5′-GTGACATAGCAGACGTAGGGAGG-3′. LIX1L KO mice were backcrossed to a C57B6/J background to avoid genetic drift and wide genomic off-target effects of Crispr/Cas9 targeting. To avoid interanimal variability that would be introduced due to differences in the stage of estrus in female mice, only male mice were used. All randomly assigned experimental controls were littermate sex- and age-matched mice. However, *Lix1l*$^{-/-}$ mice were viable and fertile without any apparent phenotypic changes. Male LIX1L knockout (KO) mice and wild-type (WT) littermate controls in C57BL/6J

background were used for this study. Animal studies are reported in compliance with the ARRIVE guidelines. All mice received human care and animal experiments were approved by the University Committee on Use and Care of Animals of the China Pharmaceutical University (Nanjing, China) (Approval NO.20200701).

For in vivo gene transfer, mice were received a single tail vein injection of 10^{11} genome copies of AAV vectors from Hanbio (Shanghai, China). HBAAV2/9-CMV-Control, HBAAV2/9-CMV-LIX1L-FALG. After injection, mice were allowed 3 weeks of recovery for subsequent experiments.

Mouse model of CCl_4 and BDL-induced hepatic fibrosis

Mouse chronic liver fibrosis was induced by intraperitoneal injected of CCl_4 (0.25mL/kg body weight) or vehicle (olive oil) three times per week for 4 weeks and was sacrificed 2 days after the last injection. The mice subjected to BDL were anesthetized with isoflurane through inhalation. The common bile duct was ligated twice with 6.0 silk sutures and cut through between the ligations. Sham-operated mice were subjected to laparotomy without BDL. The mice which received BDL or sham operation were sacrificed 14 days later. Mouse acute hepatitis was induced by a single injection of CCl_4 for 24, 48, 72h or BDL for 3 days. The mouse livers and serum were collected for subsequent experiments.

Liver histological and immunohistological staining

Liver specimens were fixed in 10% neutral buffered formalin, embedded in paraffin

and cut into 4μm sections. Next, the specimens were deparaffinized, hydrated and stained by standard methods. To examine hepatic morphology and assess liver fibrosis, H&E, Sirius Red and Masson's trichrome staining were performed, respectively. Tissue sections were immunostained for α-SMA (Abcam, ab7817), F4/80 (Abcam, ab6640), and CCL20 (Abcam, ab9829), the sections were scanned, and the images were then digitalized.

Primary mouse liver cell isolation and culture

Primary murine hepatocytes, Kupffer cells, and HSCs were isolated from the livers of male C57BL/6J mice or *Lix1l*$^{-/-}$ mice aged 6-10 weeks according to a reported protocol that includes the following steps: in situ pronase/collagenase perfusion of mouse liver, perfused livers were minced, filtered through 70 μM cell strainer (BD Bioscience), and centrifuged at 50 g for 3 min to separate hepatocytes. Hepatocytes were resuspended in Dulbecco's modified Eagle's medium (DMEM) supplemented with 10% FBS. HSCs were isolated according to the previously published method,[14] the supernatant was further centrifuged at 500 g for 10 min, resuspended in density gradient-based Nycodenz, and centrifuged at 1400 g for 17 min. HSCs were collected from the interface. Kupffer cell isolation was done according to the previously established method.[15] For isolation of Kupffer cells, hepatocytes were removed and the supernatant was centrifuged for 10 min at 500 g. The pellet was then resuspended in 25% percoll, 50% percoll (Yesen) and HBSS (Thermo Scientific). After centrifugation at 1400 g for 17 min, the Kupffer cell enriched layer was harvested from the 25% and 50% percoll

interphase, the layer of 25%-50% percoll contains KCs and LSECs fraction. As LSEC poorly attach culture plastic dish, LSECs are removed from KCs fraction by selective adherence[16].

Quantitative RT-PCR

Total RNA was extracted from the liver tissues of the mice or LX-2 cells using Tripure reagent (Roche Diagnostics, Indianapolis, IN) as described by the manufacturer. cDNA synthesis was carried out with HiScript® II Q Select RT SuperMix for qPCR (Vazyme). Quantitative PCR was performed in biological triplicates using SYBR Green reagent (Vazyme). The level of *GAPDH* (human) or *β-actin* (mouse) RNA expression was used to normalize the data. PCR primer sequences are listed in Supplementary Table 1. A melting curve of each amplicon was determined to verify its specificity.

Immunofluorescence

Cultured primary HSCs

Primary murine HSCs were seeded onto chamber slides (Nunc). Cells were culture for 3, 6, 9, 12d. Media were aspirated and cells were washed twice with PBS (Gibco) and fixed in 10% buffered formalin at room temperature for 15 min. Fixative was removed and cells were washed three times with PBS. The slides were then blocked with 3% BSA and incubated with mouse α-SMA (Abcam, ab7817,1:100) and rabbit LIX1L (Abcam, ab112952,1:100) antibodies overnight at 4 °C. After washing, the slides were incubated for 2 hours with Alexa Fluor 594 Goat anti-Mouse IgG (Invitrogen, A-11032, 1: 500), Alexa Fluor 488 Goat anti-Rabbit IgG (Invitrogen, A-11034, 1: 500). Finally,

the slides were analyzed for fluorescence using confocal laser scanning microscope (Leica, Wetzlar, Germany). Nuclear counterstaining was performed using DAPI.

Human liver tissue array

Formalin fixed and paraffin embedded liver fibrosis tissue arrays were purchased from US Biomax (LV805b) (Rockville, MD). Paraffin embedded liver fibrosis tissues were fixed in acetone for immunofluorescence then stained with the indicated antibodies: anti-LIX1L (Abcam, ab112952, 1: 50), α-SMA (Abcam, ab7817, 1: 50), Goat anti-Mouse IgG conjugated to Alexa Fluor 594 (Invitrogen, A-11032, 1: 500), Goat anti-Rabbit IgG conjugated to Alexa Fluor 488 (Invitrogen, A-11034, 1: 500). The sections were stained with DAPI and examined with a confocal laser scanning microscope (Leica, Wetzlar, Germany). The liver fibrosis stage was assessed by Ishak scale. Tissue array patient information is shown in Supplementary Table 2.

Mouse liver sections

Immunofluorescent staining was performed in formalin fixed and paraffin embedded mouse liver tissues. Paraffin embedded liver fibrosis tissues were fixed in acetone for immunofluorescence then stained with the indicated antibodies: anti-LIX1L (Abcam, ab112952, 1: 50), α-SMA (Abcam, ab7817, 1: 50), F4/80(Abcam, ab6640, 1: 500). Goat anti-Mouse IgG conjugated to Alexa Fluor 594 (Invitrogen, A-11032, 1: 500), Goat anti-Rabbit IgG conjugated to Alexa Fluor 488 (Invitrogen, A-11034, 1: 500). The sections were stained with DAPI and examined with a confocal laser scanning microscope (Leica, Wetzlar, Germany).

Cell proliferation and apoptosis assays

LX-2 cells were transfected with LIX1L siRNAs using Lipofectamine RNAiMax transfection reagent (Invitrogen). CCK8 assay was performed at different times (from 0 to 72h). Apoptosis was quantified using the FITC Annexin V Apoptosis Detection Kit (BD Biosciences Pharmingen). Briefly, after transfection with LIX1L siRNAs, LX-2 cells were harvested and suspended in Annexin-binding buffer. Subsequently, cells were incubated with Annexin V-FITC and PI for 15 min at room temperature in the dark and immediately analyzed using BD FACS Calibur flowcytometer (Becton & Dickinson Company, Franklin Lakes, NJ).

LX-2 cells culture and transfection

LX-2 cells were received as a generous gift from Hongwei He (Peking Union Medical College, Beijing). Cell lines were routinely tested for mycoplasma. All transfection procedures were carried out at a concentration of 150 nM of indicated siRNAs (RiboBio Co., LTD., Guangzhou, China) using Lipofectamine RNAiMax transfection reagent (Invitrogen). Transfected cells were cultured without perturbation for at least 48 hours before terminal assays. LX-2 cells were transiently transfected with pCMV-tag-3B-LIX1L for the over-expression of LIX1L. After transfection for 48h, the cells were treated as indicated methods then assessed.

RNA-Seq and Data Analysis.

LX-2 cells were transfected with Control or LIX1L siRNAs, and subsequently cultured for 48h, the total RNA was isolated using TRIzol (Invitrogen) reagent and RNeasy mini kit (Qiagen). RNA-seq of transfected LX-2 cells was accomplished with the assistance

of Shanghai Personal Biotechnology Co., Ltd. (Shanghai, China). P values were computed using the Benjamini–Hochburg method. The threshold we used to screen up- or downregulated mRNAs was fold change >2 and p values <0.05. The transcriptome sequencing data have been deposited in NCBI Gene Expression Omnibus (GEO) under the following accession number: GSE133121.

Chemokine and serum biochemical analysis

LX-2 cells were transfected with siControl or siLIX1L as described above. Media supernatants were harvested after 48 hours and cleared by centrifugation. The ELISA for CCL-20 was performed using the CCL20/MIP-3 alpha ELISA kit from Novus Biologicals (Littleton, Colorado, USA) following the manufacturer's instructions. CCL20 secretion was measured in supernatant of primary KCs and HSCs from WT or *Lix1l*$^{-/-}$ mice. Media supernatants were harvested after 48 hours and cleared by centrifugation. The ELISA for CCL-20 was performed using the CCL20/MIP-3 alpha ELISA kit from R&D Systems (Minneapolis, Minnesota, USA) following the manufacturer's instructions. Serum levels of alanine aminotransferase (ALT) and aspartate aminotransferase (AST) were measured using standard enzymatic procedures according to the manufactures' instruction (Nanjing Jiancheng Bioengineering Institute, Nanjing, China).

Western blot analysis

Proteins were extracted from the liver tissues or HSCs in the lysis buffer consisting of protease inhibitor cocktail tablets (MCE, IN). The extracted proteins were separated by

polyacrylamide SDS gel and electrophoretically transferred onto polyvinylidene fluoride membranes (Bio-Rad). The membranes were probed with the indicated antibodies over night at 4℃. Antibodies used in western blot were: LIX1L (Abcam, ab112952, 1: 100 dilution), α-SMA (Abcam, ab7817, 1:1000 dilution), COL1A1 (Abcam, ab34710, 1: 1,000 dilution), ALBUMIN (Abcam, ab207327, 1: 1,000 dilution), F4/80(Abcam, ab6640, 1: 500 dilution),anti-β-actin (Yesen, 30101ES60, 1: 1,000 dilution). Membranes were then incubated with a horseradish peroxidase coupled secondary antibody. Detection was performed using a LumiGLO chemiluminescent substrate system (TransGen Biotech, Beijing, China). The relative expressions were quantified densitometrically using the Image Lab Works 4.0 software, and calculated according to the reference bands of anti-β-actin.

RNA immunoprecipitation analysis

Ribonucleoprotein immunoprecipitation (RIP) were performed as described. LX-2 cells were transfected with empty vector or myc-LIX1L plasmid for 48 h. Whole-cell extracts (WCE) prepared in lysis buffer (Cell Signaling Technology, Danvers, MA) were incubated on ice for 30 minutes, followed by centrifugation at 10,000g for 15 minutes at 4 °C. The supernatants were incubated with antibodies that recognized myc-tag (CST), or with control IgG (Santa Cruz Biotechnology) for 1 hour, followed by addition of protein A/G beads (40 μL) and overnight incubation at 4 °C. Beads were washed with RIP buffer (150mM of KCl, 25mM of Tris [pH7.4], 5mM of ethylenediaminetetraacetic acid, 0.5mM DTT, and 0.5% NP-40), complexes were

treated with 20 units of RNase-free DNase I (15 minutes at 37 °C) and incubated with 0.1% sodium dodecyl sulfate and 0.5mg ml^{-1} Proteinase K (15 min at 55 °C) to remove DNA and proteins, respectively. RNA was isolated from IP by PureLink RNA mini kit (Invitrogen) and quantified by reverse transcription and qPCR (primer sequences are listed in supplementary Table 1).

RNA pull-down analysis

Biotin-labelled *CCL20* RNA transcripts were synthesized by in vitro transcription using a DNA template that included the T7 promoter according to the instructions for a Ribo™ RNAmax-T7 Transcription Kit (RiboBio, Guangzhou, China). PCR fragments were amplified with forward primers containing T7 RNA polymerase promoter sequences. Purified PCR products were used as a DNA template for in vitro transcription. Next, 1 mg of lysates from whole cells of LX-2 was incubated with 3 µg of purified biotinylated transcripts for 1 h at 25 °C. The complexes were isolated with streptavidin agarose beads (Invitrogen). The beads were washed briefly three times and boiled in sodium dodecyl sulfate (SDS) buffer, and the retrieved protein was detected using the standard western blot technique.

Bone marrow transplantation.

Bone marrow transplantation (BMT) was performed as described previously.[4] Mouse macrophages were depleted by injection of liposomal clodronate (200 µl intravenously), followed by lethal irradiation with 9-10 Gy to deplete Kupffer cells and accelerate macrophage turnover. Then we intravenous injection of 1 x 10^7 bone marrow (BM)

cells from donor mice. Mice were allowed to recuperate for 6-8 weeks prior to induction of liver fibrosis[17].

Statistical analysis.

The results are presented as mean ± SEM. Statistical differences between two groups were analyzed by the unpaired Student's t test with a two-tailed distribution. Differences between multiple groups of data were analyzed by one-way ANOVA with Bonferroni correction (Graph Pad Prism 8.0, San Diego, CA, USA). P-values less than 0.05 were considered statistically significant.

References

1 Mao, Y., Kucuk, B. & Irvine, K. D. Drosophila lowfat, a novel modulator of Fat signaling. *Development* **136**, 3223-3233, doi:10.1242/dev.036152 (2009).

2 Lee, Y. A., Wallace, M. C. & Friedman, S. L. Pathobiology of liver fibrosis: a translational success story. *Gut* **64**, 830-841, doi:10.1136/gutjnl-2014-306842 (2015).

3 Wasmuth, H. E., Tacke, F. & Trautwein, C. Chemokines in liver inflammation and fibrosis. *Semin Liver Dis* **30**, 215-225, doi:10.1055/s-0030-1255351 (2010).

4 Liu, Y. *et al.* Activated hepatic stellate cells directly induce pathogenic Th17 cells in chronic hepatitis B virus infection. *Exp Cell Res* **359**, 129-137, doi:10.1016/j.yexcr.2017.08.001 (2017).

5 Brennan, C. M. & Steitz, J. A. HuR and mRNA stability. *Cell Mol Life Sci* **58**, 266-277, doi:10.1007/PL00000854 (2001).

6 Frevel, M. A. *et al.* p38 Mitogen-activated protein kinase-dependent and -independent signaling of mRNA stability of AU-rich element-containing transcripts. *Mol Cell Biol* **23**, 425-436, doi:10.1128/mcb.23.2.425-436.2003 (2003).

7 De Minicis, S. *et al.* Gene expression profiles during hepatic stellate cell activation in culture and in vivo. *Gastroenterology* **132**, 1937-1946, doi:10.1053/j.gastro.2007.02.033 (2007).

8 Bonacchi, A. *et al.* The chemokine CCL21 modulates lymphocyte recruitment and fibrosis in chronic hepatitis C. *Gastroenterology* **125**, 1060-1076 (2003).

9 Sprenger, H. *et al.* Induction of neutrophil-attracting chemokines in transforming rat hepatic stellate cells. *Gastroenterology* **113**, 277-285 (1997).

10 Duffield, J. S. *et al.* Selective depletion of macrophages reveals distinct, opposing roles during liver injury and repair. *J Clin Invest* **115**, 56-65, doi:10.1172/JCI22675 (2005).

11 Affo, S. *et al.* CCL20 mediates lipopolysaccharide induced liver injury and is a potential driver of inflammation and fibrosis in alcoholic hepatitis. *Gut* **63**, 1782-1792, doi:10.1136/gutjnl-2013-306098 (2014).

12 Schutyser, E., Struyf, S. & Van Damme, J. The CC chemokine CCL20 and its receptor CCR6. *Cytokine Growth Factor Rev* **14**, 409-426 (2003).

13 Tacke, F. & Zimmermann, H. W. Macrophage heterogeneity in liver injury and fibrosis. *Journal of hepatology* **60**, 1090-1096, doi:10.1016/j.jhep.2013.12.025 (2014).

14 Koo, J. H., Lee, H. J., Kim, W. & Kim, S. G. Endoplasmic Reticulum Stress in Hepatic Stellate Cells Promotes Liver Fibrosis via PERK-Mediated Degradation of HNRNPA1 and Up-regulation of SMAD2. *Gastroenterology* **150**, 181-193 e188, doi:10.1053/j.gastro.2015.09.039 (2016).

15 Wang, J. *et al.* Sirtuin 2 aggravates postischemic liver injury by deacetylating mitogen-activated protein kinase phosphatase-1. *Hepatology* **65**, 225-236, doi:10.1002/hep.28777 (2017).

16 Lan, T. *et al.* Sphingosine kinase 1 promotes liver fibrosis by preventing miR-19b-3p-mediated inhibition of CCR2. *Hepatology* **68**, 1070-1086, doi:10.1002/hep.29885 (2018).

17 Kisseleva, T. *et al.* Bone marrow-derived fibrocytes participate in pathogenesis of liver fibrosis. *J Hepatol* **45**, 429-438, doi:10.1016/j.jhep.2006.04.014 (2006).

Supplemental Table 1. Primer and siRNA Sequences sequences used in this study.

**Primers used for qRT-PCR**		
**Genes**	**Forward primer (5'-3')**	**Reverse primer (5'-3')**
human		
LIX1L	CGGACCTTATAGACGACAGCTC	GTGCCTTCCGATTCATGCTG
COL1A1	GTGCGATGACGTGATCTGTGA	CGGTGGTTTCTTGGTCGGT
α-SMA	CCTTGTTTGGGAAGCAAGTGG	TGGAGCTGCTTCACAGGATT
TGFβ1	GGCCAGATCCTGTCCAAGC	GTGGGTTTCCACCATTAGCAC
PDGF	CTCGATCCGCTCCTTTGATGA	CGTTGGTGCGGTCTATGAG
TIMP-1	CTTCTGCAATTCCGACCTCGT	ACGCTGGTATAAGGTGGTCTG
CCL20	AGCCCAAGAACAGAAAGAACCT	TTGGACAAGTCCAGTGAGGC
CCR6	AAGAGAGGGCCCACGTGTA	GAAGGAGCTGTCTGTTCCACA
IL-1β	TTCGAGGCACAAGGCACAA	CCATCATTTCACTGGCGAGC
TNF-α	GAGGCCAAGCCCTGGTATG	CGGGCCGATTGATCTCAGC
CCL2	CAGCCAGATGCAATCAATGCC	TGGAATCCTGAACCCACTTCT
CXCL2	TGAGCCTCAAAGCAACCTCA	ATCAGCGAGTCTCTTCTTCCCT
CXCL3	CGCCCAAACCGAAGTCATAG	GCTCCCCTTGTTCAGTATCTTTT
CXCL6	AGAGCTGCGTTGCACTTGTT	GCAGTTTACCAATCGTTTTGGGG
CCR10	GCAAACGCAAGGATGTCGC	CGTAGAGAACGGGATTGAGGC
GADPH	GACCTGCCGTCTAGAAAAAC	TTGAAGTCAGAGGAGACCAC
mouse		
Lix1l	GGCTACGGTCGAGTGAATGTG	GAGCCCCGTTCTTCAAGTCAG
α-sma	CCCAGACATCAGGGAGTAATGG	TCTATCGGATACTTCAGCGTCA
Col1a1	TAAGGGTCCCCAATGGTGAGA	GGGTCCCTCGACTCCTACAT
Tgfβ1	CTCCCGTGGCTTCTAGTGC	GCCTTAGTTTGGACAGGATCTG
Pdgf	CATCCGCTCCTTTGATGATCTT	GTGCTCGGGTCATGTTCAAGT
Timp1	GCAACTCGGACCTGGTCATAA	CGGCCCGTGATGAGAAACT
Ccl20	CGACTGTTGCCTCTCGTACA	GAGGAGGTTCACAGCCCTTT
Ccr6	TTGTGTCTGTCAACAGAATAGTCCT	GGAATTCATTCCCCAGAAAATGCT
Il1β	GCAACTGTTCCTGAACTCAACT	ATCTTTTGGGGTCCGTCAACT
Tnfα	CCCTCACACTCAGATCATCTTCT	GCTACGACGTGGGCTACAG
Ccl2	TTAAAAACCTGGATCGGAACCAA	GCATTAGCTTCAGATTTACGGGT
Cxcl2	CCAACCACCAGGCTACAGG	GCGTCACACTCAAGCTCTG
Cxcl3	AGTGGTGCTTTGTTTGCACG	ACACATCCAGACACCGTTGG
Cxcl6	TCCAGCTCGCCATTCATGC	TTGCGGCTATGACTGAGGAAG
Ccr10	GGACTTTACTCCGGGTACGAT	CAGGGAGACACTGGGTTGGA
β-actin	GGCTGTATTCCCCTCCATCG	CCAGTTGGTAACAATGCCATGT
**Primers used for RIP-qPCR**		
CCL20	AGTTGTCTGTGTGCGCAAAT	CCCTCCATGATGTGCAAGTG
**Primers used for RNA pulldown**		
CCL20	GGACTCTAATACGACTCACTATAGG	GGTTTTTAGCTCAAAGAACAG

5’UTR	GAGAATATAACAGCACTCCCA (T7)	
CCL20 CDS	GGACTCTAATACGACTCACTATAGGGATGTGCTGTACCAAGAG (T7)	CATGTTCTTGACTTTTTTACTGAGG
CCL20 3’UTR	GGACTCGTAATACGACTCACTATAGGGAAACTGTGGCTTTTCTGG (T7)	TTTTTTTTAACAGAAGAACTTTTTG

**Sequence for siRNA**	
**Name**	
LIX1L-siRNA1#	CCCGAAGAATCACTGATGA
LIX1L-siRNA2#	GAGGAGTGCTGCAAAGATT
CCL20-siRNA1#	ACCGTATTCTTCATCCTAA
CCL20-siRNA2#	GACTGCTGTCTTGGATACA

Supplementary Table 2. Tissue array patient information

No.	Age	Sex	Organ	Pathology diagnosis	Type	Tissue ID.
1	34	M	Liver	Adjacent normal liver tissue	NAT	Dlv030943
2	35	M	Liver	Adjacent normal liver tissue	NAT	Dlv030221
3	64	M	Liver	Adjacent normal liver tissue	NAT	Dlv062184
4	38	M	Liver	Cancer adjacent liver tissue	AT	Dlv060420
5	27	M	Liver	Adjacent normal liver tissue	NAT	Dlv031249
6	52	F	Liver	Adjacent normal liver tissue	NAT	Dlv010201
7	41	M	Liver	Adjacent normal liver tissue	NAT	Dlv031997
8	42	F	Liver	Adjacent normal liver tissue	NAT	Dlv031507
9	38	F	Liver	Adjacent normal liver tissue	NAT	Dlv030490
10	25	F	Liver	Adjacent normal liver tissue	NAT	Dlv031716
11	35	M	Liver	Adjacent normal liver tissue	NAT	Dlv031809
12	56	M	Liver	Cancer adjacent liver tissue	AT	Dlv041091
13	49	M	Liver	Cancer adjacent liver tissue	AT	Dlv051141
14	51	F	Liver	Adjacent normal liver tissue	NAT	Dlv061716
15	65	F	Liver	Adjacent normal liver tissue	NAT	Dlv051119
16	56	M	Liver	Adjacent normal liver tissue	NAT	Dlv040994
17	63	M	Liver	Adjacent normal liver tissue	NAT	Dlv031797
18	35	F	Liver	Adjacent normal liver tissue	NAT	Dlv060974
19	61	F	Liver	Adjacent normal liver tissue	NAT	Dlv041048
20	73	M	Liver	Cancer adjacent liver tissue	AT	Dlv041067
21	32	M	Liver	Adjacent normal liver tissue	NAT	Dlv041167
22	35	M	Liver	Adjacent normal liver tissue	NAT	Dlv05N006
23	40	M	Liver	Adjacent normal liver tissue	NAT	Dlv05N013
24	38	M	Liver	Adjacent normal liver tissue	NAT	Dlv05N015
25	40	M	Liver	Adjacent normal liver tissue	NAT	Dlv05N014
26	16	M	Liver	Adjacent normal liver tissue	NAT	Dlv06N007
27	45	M	Liver	Adjacent normal liver tissue	NAT	Dlv06N001
28	35	F	Liver	Adjacent normal liver tissue	NAT	Dlv03N009
29	21	F	Liver	Adjacent normal liver tissue	NAT	Dlv05N001
30	50	F	Liver	Adjacent normal liver tissue	NAT	Dlv03N005
31	45	M	Liver	Chronic hepatitis	Inflammation	Dlv030361
32	43	M	Liver	Chronic hepatitis	Inflammation	Dlv022879
33	47	F	Liver	Chronic hepatitis with fatty degeneration	Inflammation	Dlv024176
34	41	M	Liver	Chronic hepatitis	Inflammation	Dlv030358
35	59	F	Liver	Chronic hepatitis	Inflammation	Dlv031753
36	50	M	Liver	Chronic hepatitis	Inflammation	Dlv031874
37	51	M	Liver	Chronic hepatitis	Inflammation	Dlv021087
38	58	M	Liver	Chronic hepatitis	Inflammation	Dlv051217
39	50	M	Liver	Chronic hepatitis	Inflammation	Dlv041081
40	65	M	Liver	Chronic hepatitis	Inflammation	Dlv040078

41	49	M	Liver	Cirrhosis	Cirrhosis	Dlv031740
42	42	M	Liver	Cirrhosis	Cirrhosis	Dlv010124
43	43	M	Liver	Cirrhosis	Cirrhosis	Dlv010288
44	48	M	Liver	Cirrhosis (portal area hyperplasia)	Cirrhosis	Dlv031031
45	67	M	Liver	Cirrhosis	Cirrhosis	Dlv010874
46	57	M	Liver	Cirrhosis	Cirrhosis	Dlv011339
47	33	M	Liver	Cirrhosis	Cirrhosis	Dlv031799
48	55	M	Liver	Cirrhosis	Cirrhosis	Dlv022056
49	39	M	Liver	Cirrhosis	Cirrhosis	Dlv041496
50	38	M	Liver	Cirrhosis	Cirrhosis	Dlv022086
51	51	M	Liver	Cirrhosis	Cirrhosis	Dlv022101
52	34	M	Liver	Cirrhosis	Cirrhosis	Dlv041727
53	48	M	Liver	Cirrhosis	Cirrhosis	Dlv022148
54	48	M	Liver	Cirrhosis	Cirrhosis	Dlv022151
55	40	M	Liver	Cirrhosis	Cirrhosis	Dlv022889
56	40	M	Liver	Cirrhosis	Cirrhosis	Dlv022892
57	46	M	Liver	Cirrhosis	Cirrhosis	Dlv023412
58	39	M	Liver	Cirrhosis	Cirrhosis	Dlv023420
59	51	M	Liver	Cirrhosis	Cirrhosis	Dlv023640
60	51	M	Liver	Cirrhosis	Cirrhosis	Dlv023644
61	35	M	Liver	Cirrhosis	Cirrhosis	Dlv023651
62	39	F	Liver	Cirrhosis	Cirrhosis	Dlv023921
63	48	M	Liver	Cirrhosis	Cirrhosis	Dlv023927
64	45	F	Liver	Cirrhosis	Cirrhosis	Dlv030258
65	31	M	Liver	Cirrhosis	Cirrhosis	Dlv031817
66	40	M	Liver	Cirrhosis	Cirrhosis	Dlv024196
67	60	M	Liver	Cirrhosis	Cirrhosis	Dlv024197
68	68	F	Liver	Cirrhosis	Cirrhosis	Dlv024306
69	51	M	Liver	Cirrhosis	Cirrhosis	Dlv024312
70	47	M	Liver	Cirrhosis	Cirrhosis	Dlv024484
71	57	M	Liver	Cirrhosis	Cirrhosis	Dlv062670
72	49	M	Liver	Cirrhosis	Cirrhosis	Dlv030086
73	57	M	Liver	Cirrhosis	Cirrhosis	Dlv050119
74	47	M	Liver	Cirrhosis	Cirrhosis	Dlv030194
75	23	M	Liver	Cirrhosis	Cirrhosis	Dlv030200
76	62	F	Liver	Cirrhosis	Cirrhosis	Dlv030209
77	58	M	Liver	Cirrhosis	Cirrhosis	Dlv051446
78	60	M	Liver	Cirrhosis	Cirrhosis	Dlv062596
79	38	M	Liver	Cirrhosis	Cirrhosis	Dlv030270
80	53	M	Liver	Cirrhosis	Cirrhosis	Dlv060134

Supplementary Figure legends

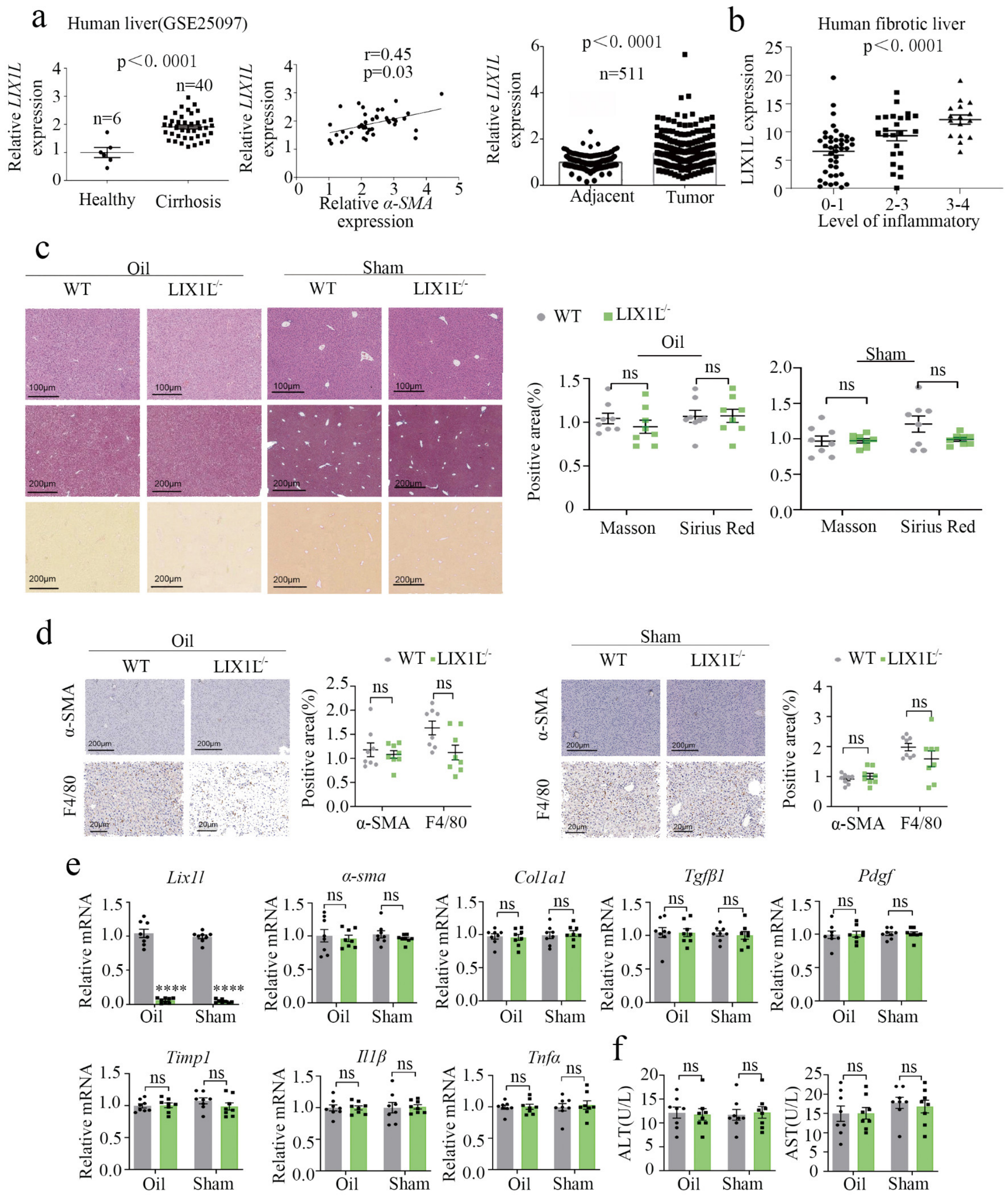

Supplementary Figure s1. (a) *LIX1L* expression was up-regulated in a large cohort of fibrosis patients ((GSE25097) (n=46) (left)). Correlation of *LIX1L* with α-SMA was analyzed, and linear regression coefficient and statistical significance are indicated (middle). *LIX1L* expression was up-regulated in HCC tumor tissues (GSE25097) (n=511) (right). (b) The positive correlation of LIX1L with the level of inflammatory in fibrosis patients from tissue arrays. (c-d) Phenotypic of WT littermates and *Lix1l*$^{-/-}$ mice with oil or sham treatment (n=8 per group). Representative images of H&E, Sirius Red, Masson's trichrome staining (c) and IHC staining for α-SMA, F4/80 from liver tissues of WT littermates and *Lix1l*$^{-/-}$ mice(d). Scale bar: 200 μm. (e) The qPCR analysis of *Lix1l* mRNA, Hepatic mRNAs of fibrogenic genes and inflammatory genes were measured by RT-qPCR assays in WT and *Lix1l*$^{-/-}$ mice. (f) Serum levels of ALT and AST in mice. Data are presented as means ± SEM. NS: non-significant. * P<0.05; NS:

non-significant.

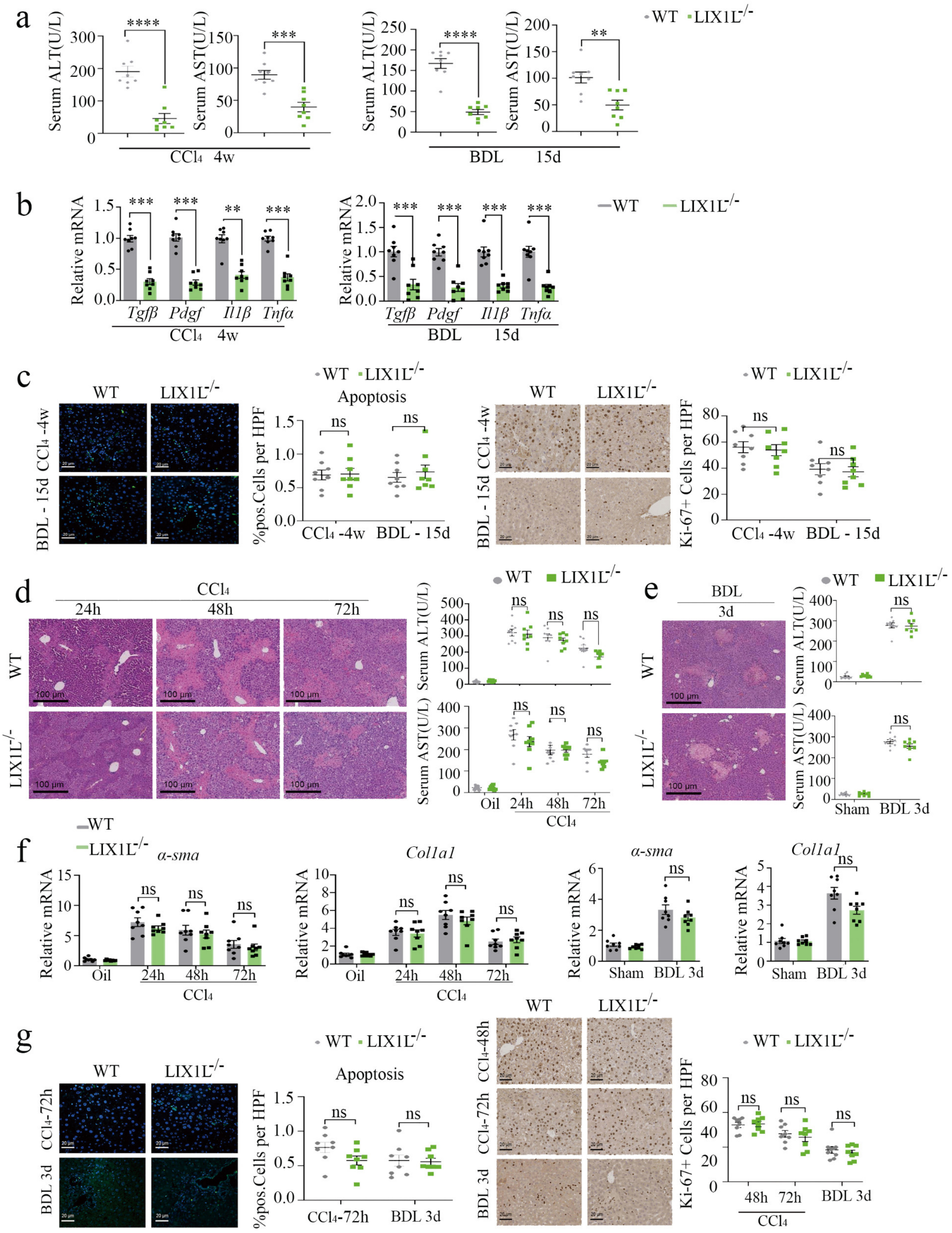

Supplementary Figure s2. (a) Serum levels of ALT and AST in WT and *Lix1l*$^{-/-}$ mice with CCl_4 or BDL treatment (n=8 per group). (b) Hepatic mRNAs of fibrogenic genes and inflammatory genes were measured by RT-qPCR assays in WT and *Lix1l*$^{-/-}$ mice with CCl_4 or BDL treatment. (c) Hepatocyte apoptosis (left) and proliferation (right) in WT mice and *Lix1l*$^{-/-}$ mice following CCL_4 for 4 weeks or BDL for 15 days treatment (n=8 per group). Immunofluorescence staining of liver sections for TUNEL (green).

Scale bar: 20 μm. Total apoptotic cells TUNEL positive was quantified (left). Immunohistochemistry staining for Ki-67. Scale bar: 20 μm. Ki-67 positive hepatocytes was quantified(right). (d) Representative images of H&E from liver tissues of WT littermates and *Lix1l*$^{-/-}$ mice treated with CCl_4. Scale bar: 100 μm (left). Serum levels of ALT and AST in mice (right). (e) Representative images of H&E from liver tissues of WT littermates and *Lix1l*$^{-/-}$ mice treated with BDL. Scale bar: 100 μm (left). Serum levels of ALT and AST in mice (right). (f) Hepatic mRNAs of fibrogenic genes. (g) Hepatocyte apoptosis (left) and proliferation (right) in WT mice and *Lix1l*$^{-/-}$ mice following CCL_4 for 48, 72h or BDL for 3 days treatment (n=8 per group). Immunofluorescence staining of liver sections for TUNEL (green). Scale bar: 20 μm. Total apoptotic cells TUNEL positive was quantified(left). Immunohistochemistry staining for Ki-67(right). Scale bar: 20 μm. Ki-67 positive hepatocytes was quantified. Data are presented as means ± SEM. NS: non-significant. * $P<0.05$; ** $P<0.01$; *** $P<0.001$; ****$P<0.0001$; NS: non-significant.

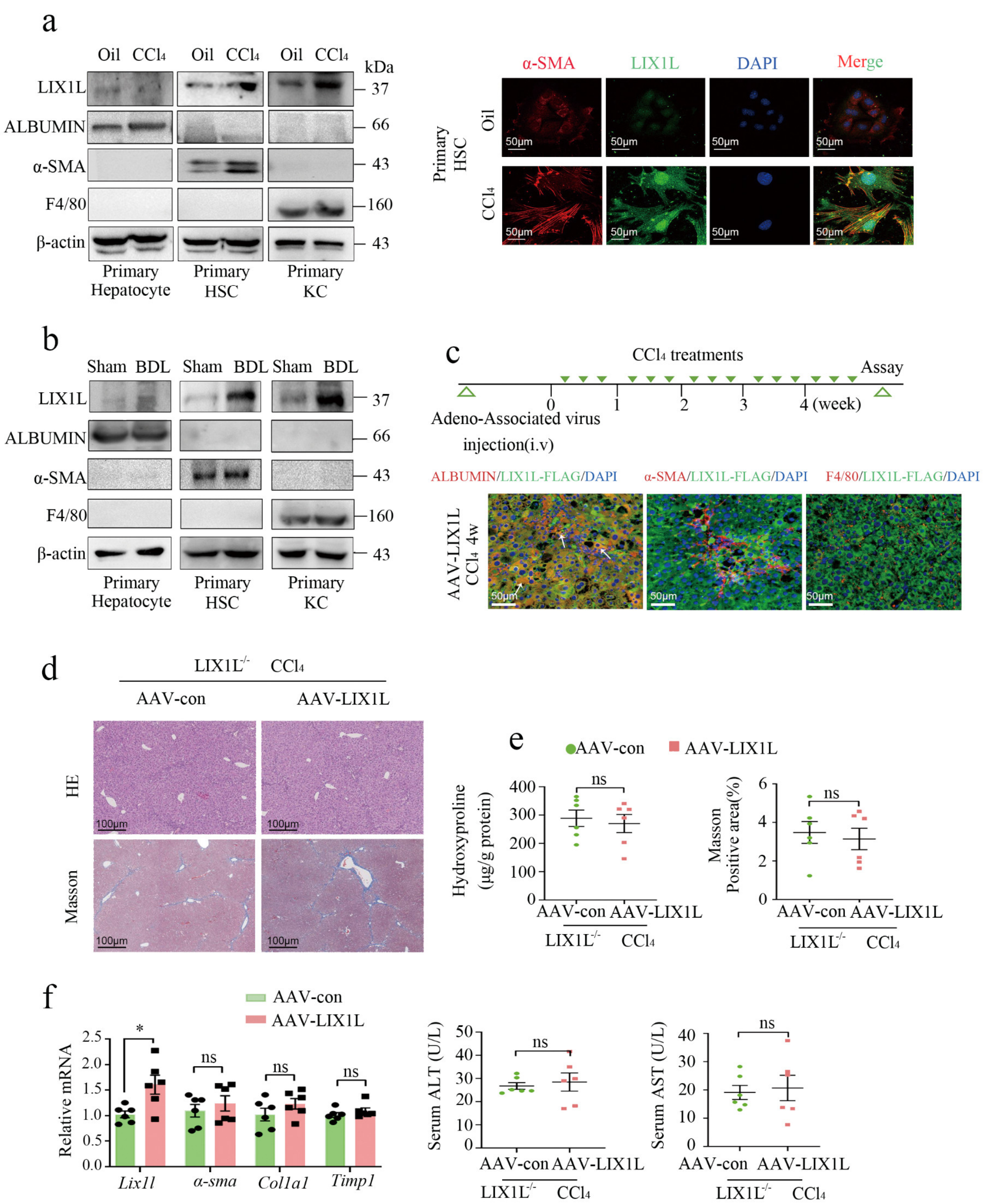

Supplementary Figure s3. (a) Immunoblotting analysis of LIX1L in isolated and cultured Hepatocytes, KCs, and 6-day cultured HSCs from mice with or without CCl_4 treatment (left). Double immunofluorescence staining for LIX1L (green) and α-SMA (red) on HSCs from WT mice treated with CCl_4 for 4 weeks (right). Nuclei were counter-stained with DAPI (blue). Scale bar: 50 μm. (b) Immunoblotting analysis of LIX1L in isolated and cultured Hepatocytes, HSCs and KCs from mice operated with sham or BDL. (c-f) Schematic overview of experiments analyzing the effect of LIX1L overexpression in CCl_4-induced *Lix1l*$^{-/-}$ mice (n=6 per group). (c) Double immunofluorescence staining for LIX1L-FLAG (green), ALBUMIN (red), α-SMA (red) and F4/80(red) from liver tissues of *Lix1l*$^{-/-}$ mice after AAV-LIX1L injection and treated with CCl_4. Nuclei were counter-stained with DAPI (blue). Scale bar: 50 μm. (d) Representative images of H&E from liver tissues of *Lix1l*$^{-/-}$ mice after AAV-LIX1L

injection and treated with CCl_4. Scale bar: 100 µm. (e) Liver fibrosis was evaluated by hydroxyproline content. (f) RT-qPCR assays fibrogenic genes (left). Serum levels of ALT and AST in mice (right). Data are presented as means ± SEM. NS: non-significant.

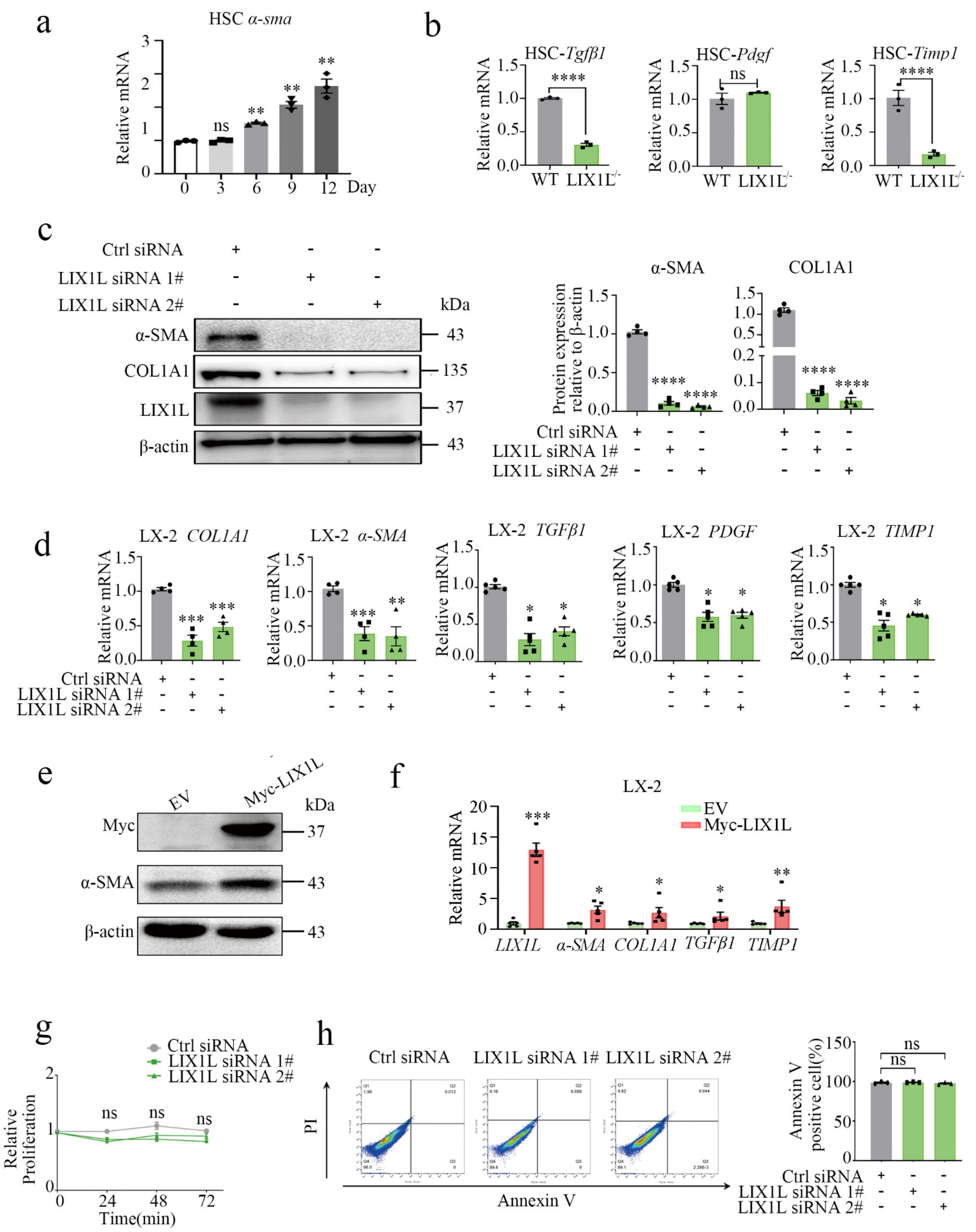

Supplementary Figure s4. (a) α-SMA mRNA expression in cultured primary HSCs from WT mice without treatment at the indicated times. (b) Fibrogenic gene expression in cultured HSCs (9 days) isolated from WT and *Lix1l*$^{-/-}$ mice without treatment. (c) Immunoblotting assays of LIX1L, α-SMA and collagen I in LX-2 cells transfected with control or LIX1L siRNAs. (d) Fibrogenic genes were measured by RT-qPCR assays in LX-2 cells transfected with control or LIX1L siRNAs. (e) LX-2 cells were transfected with LIX1L plasmid or empty vector (EV). Representative immunoblotting bands of

Myc-tag and α-SMA. (f) Fibrogenic genes were measured by RT-qPCR assays in LX-2 cells transfected with LIX1L plasmid or empty vector (EV). (g) LX-2 cells were transfected with control or LIX1L siRNAs for indicated time. CCK8 assay was carried out to detect the cell number. (h) Percentage of Annexin V positive cells in LX-2 cells transfected with control or LIX1L siRNAs for 48 hours. Data are presented as means ± SEM. * P<0.05; ** P<0.01; *** P<0.001; ****P<0.0001; NS: non-significant.

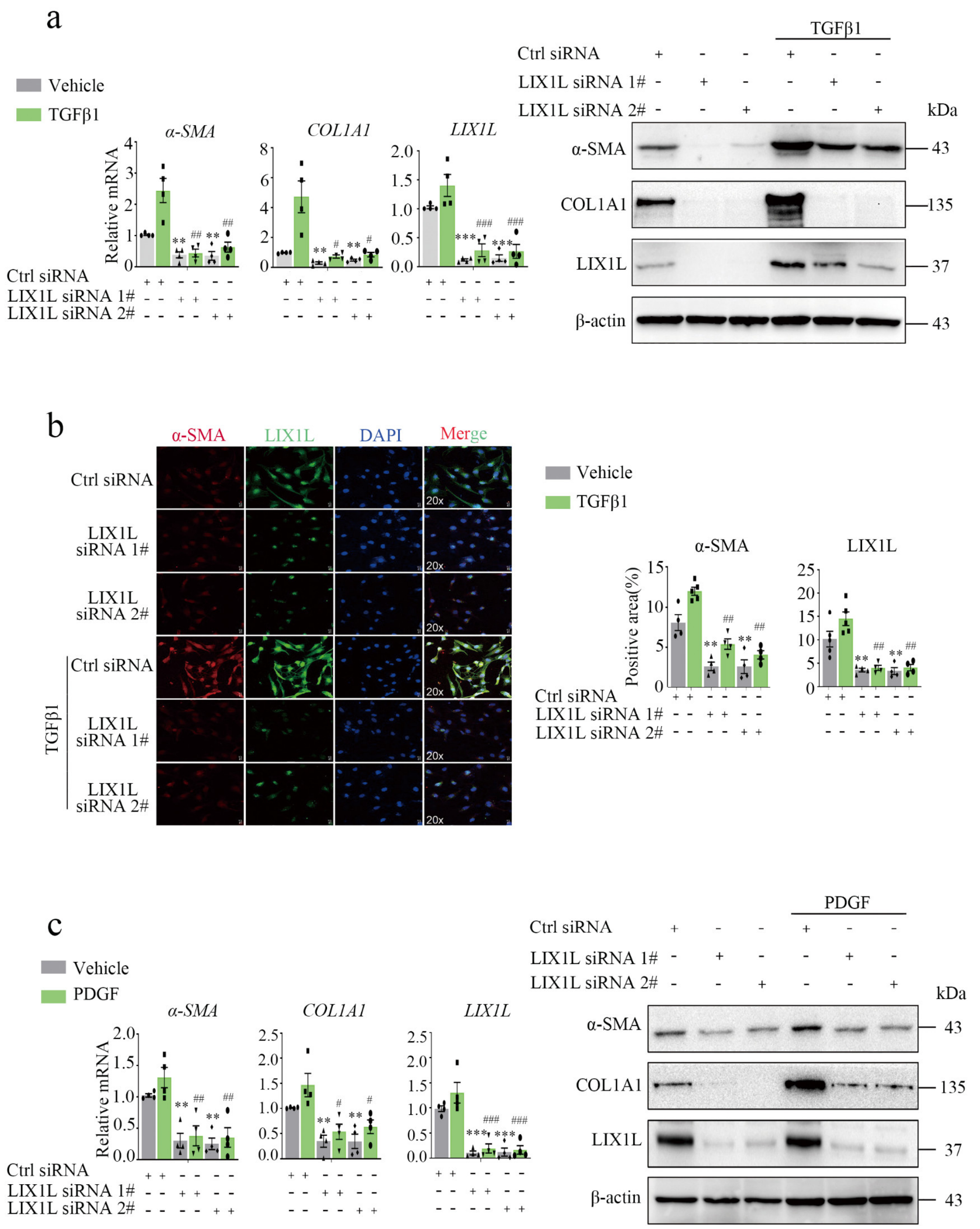

Supplementary Figure s5. (a-b) LX-2 cells were starved in DMEM without FBS and transfected with control or LIX1L siRNAs with or without 5 ng/ml TGFβ1 treatment for 6 hours. (a) Fibrogenic genes were measured by RT-qPCR assays. Immunoblotting assays of LIX1L, α-SMA and COL1A1. (b) Double immunofluorescence staining for LIX1L (green) and α-SMA (red) in LX-2 cells. The right panels show quantitative analyses of LIX1L and α-SMA expression. (c) LX-2 cells were starved in DMEM

without FBS and transfected with control or LIX1L siRNAs with or without 20 ng/ml PDGF treatment for 6 hours. Fibrogenic genes were measured by RT-qPCR assays (left). Immunoblotting assays of LIX1L, α-SMA and COL1A1(right). Data are presented as means ± SEM. * $P<0.05$; ** $P<0.01$; *** $P<0.001$ versus control without TGFβ1 or PDGF treatment, # $P<0.05$; ##$P<0.01$; ###$P<0.001$ versus control with TGFβ1 or PDGF treatment.

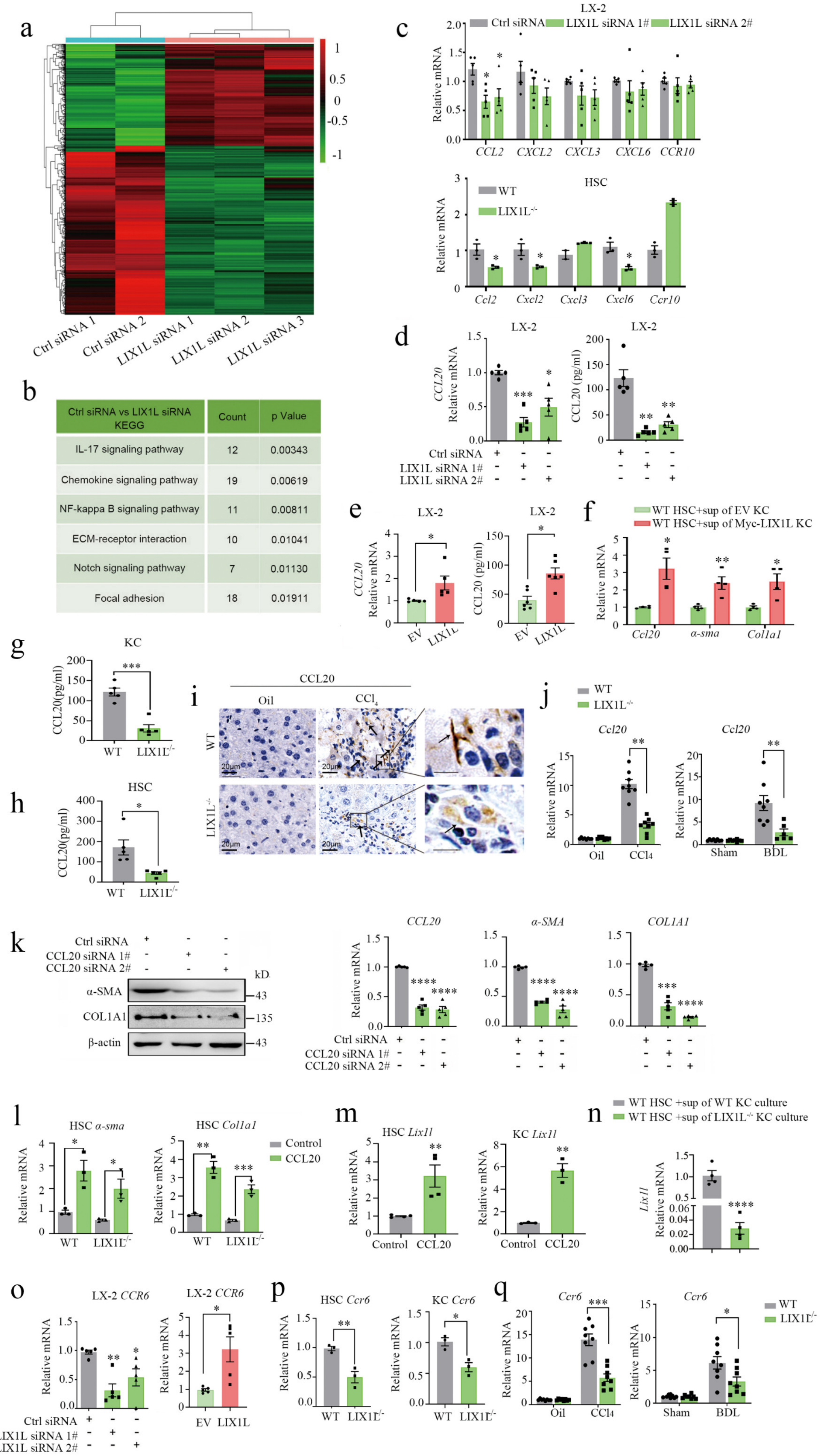
a
Ctrl siRNA 1
Ctrl siRNA 2
LIX1L siRNA 1
LIX1L siRNA 2
LIX1L siRNA 3
b
Ctrl siRNA vs LIX1L siRNA KEGG
Count
p Value
IL-17 signaling pathway 12 0.00343
Chemokine signaling pathway 19 0.00619
NF-kappa B signaling pathway 11 0.00811
ECM-receptor interaction 10 0.01041
Notch signaling pathway 7 0.01130
Focal adhesion 18 0.01911
c
LX-2
Ctrl siRNA
LIX1L siRNA 1#
LIX1L siRNA 2#
Relative mRNA
CCL2 CXCL2 CXCL3 CXCL6 CCR10
HSC
WT
LIX1L-/-
Ccl2 Cxcl2 Cxcl3 Cxcl6 Ccr10
d
LX-2
CCL20 Relative mRNA
CCL20 (pg/ml)
Ctrl siRNA
LIX1L siRNA 1#
LIX1L siRNA 2#
e
LX-2
EV LIX1L
f
WT HSC+sup of EV KC
WT HSC+sup of Myc-LIX1L KC
Ccl20 α-sma Col1a1
g
KC
CCL20(pg/ml)
WT LIX1L-/-
h
HSC
i
CCL20
Oil
CCl4
WT
LIX1L-/-
20μm
j
WT
LIX1L-/-
Ccl20
Oil CCl4
Sham BDL
k
Ctrl siRNA
CCL20 siRNA 1#
CCL20 siRNA 2#
kD
α-SMA
COL1A1
β-actin
43
135
43
CCL20
α-SMA
COL1A1
l
HSC α-sma
HSC Col1a1
Control
CCL20
m
HSC Lix1l
KC Lix1l
n
WT HSC +sup of WT KC culture
WT HSC +sup of LIX1L-/- KC culture
Lix1l Relative mRNA
o
LX-2 CCR6
p
HSC Ccr6
KC Ccr6
q
Ccr6

Supplementary Figure s6. (a) Heatmaps of gene-expression data from RNA-seq analysis. LX-2 cells were transfected with control or LIX1L siRNAs for 48h and the global gene expression change was measured by RNA-seq. (b) KEGG pathway analysis of genes affected by LIX1L. (c) RT-qPCR analyses for chemokines in LX2 cells transfected with control or LIX1L siRNAs (up), and cultured primary HSCs (3 days) isolated from WT and *Lix1l*$^{-/-}$ mice without treatment (bottom). (d-e) CCL20 expression and secretion were measured in LX-2 cells with LIX1L knockdown (d) or LIX1L overexpression (e). (f) CCL20 mRNA and fibrogenic genes expression were measured in HSCs exposed to EV or Myc-LIX1L KCs supernatant. (g, h) CCL20 secretion was measured in supernatant of cultured primary HSCs (6 days) and KCs (3 days) from WT and *Lix1l*$^{-/-}$ mice without treatment. (i) IHC staining of CCL20 in livers from WT or *Lix1l*$^{-/-}$ mice with CCl_4 treatment (n=8 per group). Scale bar: 20 μm. (j) RT-qPCR analyses for *CCL20* mRNA in livers from WT and *Lix1l*$^{-/-}$ mice with or without CCl_4 or BDL treatment. (k) Immunoblotting assays of α-SMA and collagen I in LX-2 cells transfected with control or CCL20 siRNAs (left). Fibrogenic genes were measured by RT-qPCR assays in LX-2 cells transfected with control or CCL20 siRNAs (right). (l) Primary HSCs were isolated from WT and *Lix1l*$^{-/-}$ mice and cultured for 3 day, and then treated with CCL20 (250 ng/mL) for 48h. RT-qPCR analyses for fibrogenic genes in primary HSCs. (m) Primary HSCs and KCs were isolated from WT mice and cultured for 3 day, and then treated with CCL20 (250 ng/mL) for 48h. RT-qPCR analyses for *Lix1l* in primary HSCs and KCs. (n) LIX1L mRNA was measured in HSCs with WT or *Lix1l*$^{-/-}$ KC supernatant. (o) RT-qPCR analyses for *CCR6* mRNA in LIX1L knockdown or overexpressed LX-2 cells. (p) RT-qPCR analyses for *CCR6* mRNA in isolated and cultured HSCs and KCs from WT and *Lix1l*$^{-/-}$ mice without treatment. (q) *Ccr6* mRNA was measured by RT-qPCR assay in livers from WT and *Lix1l*$^{-/-}$ mice induced by CCl_4 and BDL (n=8 per group). Data are presented as means ± SEM. * $P<0.05$; ** $P<0.01$; *** $P<0.001$.

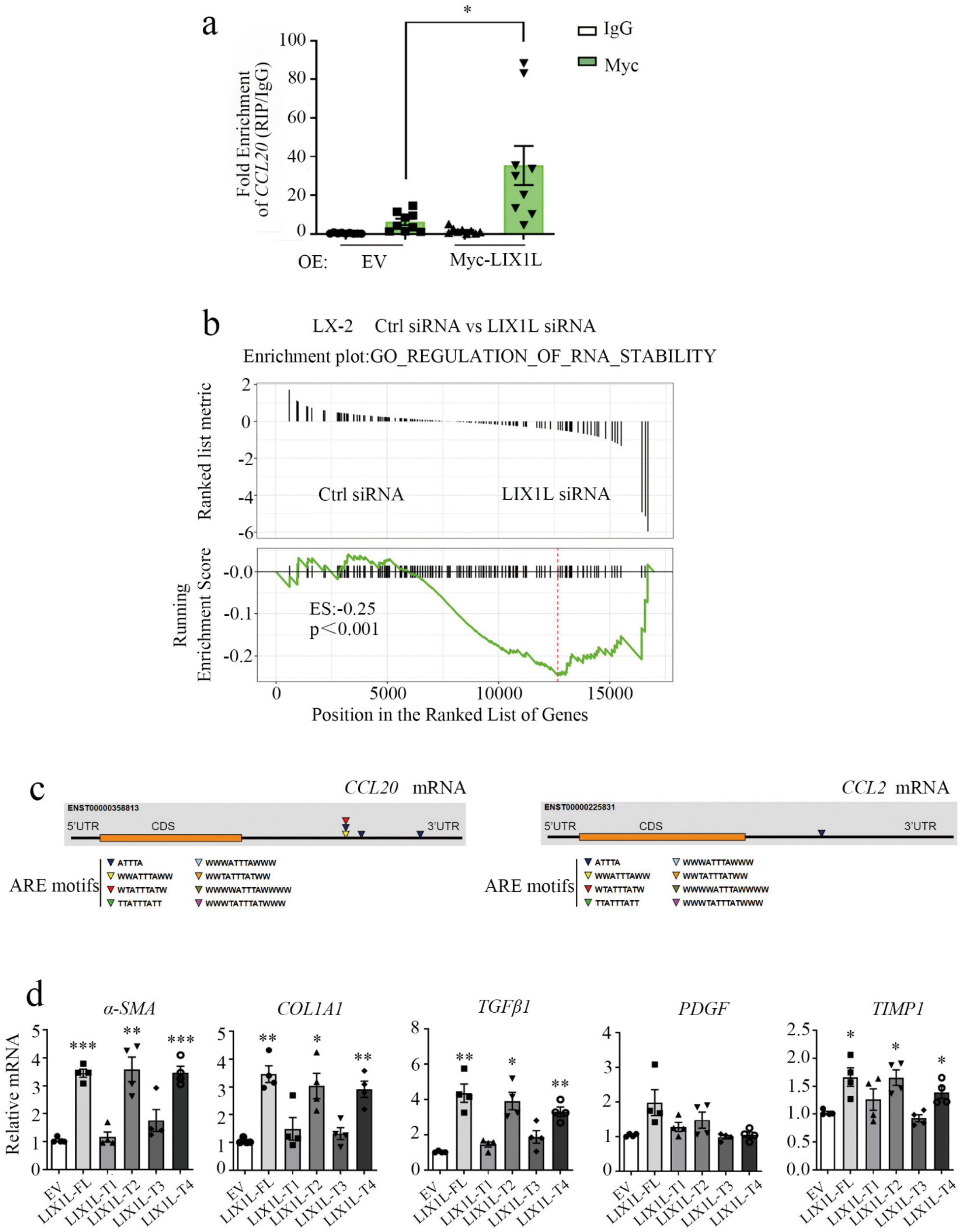

Supplementary Figure s7. (a) RIP assays to determine the interaction between *CCL20* mRNA and LIX1L employing lysates from LX-2 cells transfected with Myc-tagged LIX1L vectors and immunoprecipitated with IgG or Myc-tag antibody. (b) GSEA analysis showing that differentially expressed genes with LIX1L knockdown were enriched in RNA stability. (c) Schematic of AREs in the 3' UTR of human *CCL20* and *CCL2* mRNA. (d) Fibrogenic genes were measured by RT-qPCR assays in LX-2 cells transfected with LIX1L mutant. Data are presented as means ± SEM. * $P<0.05$; ** $P<0.01$; *** $P<0.001$.

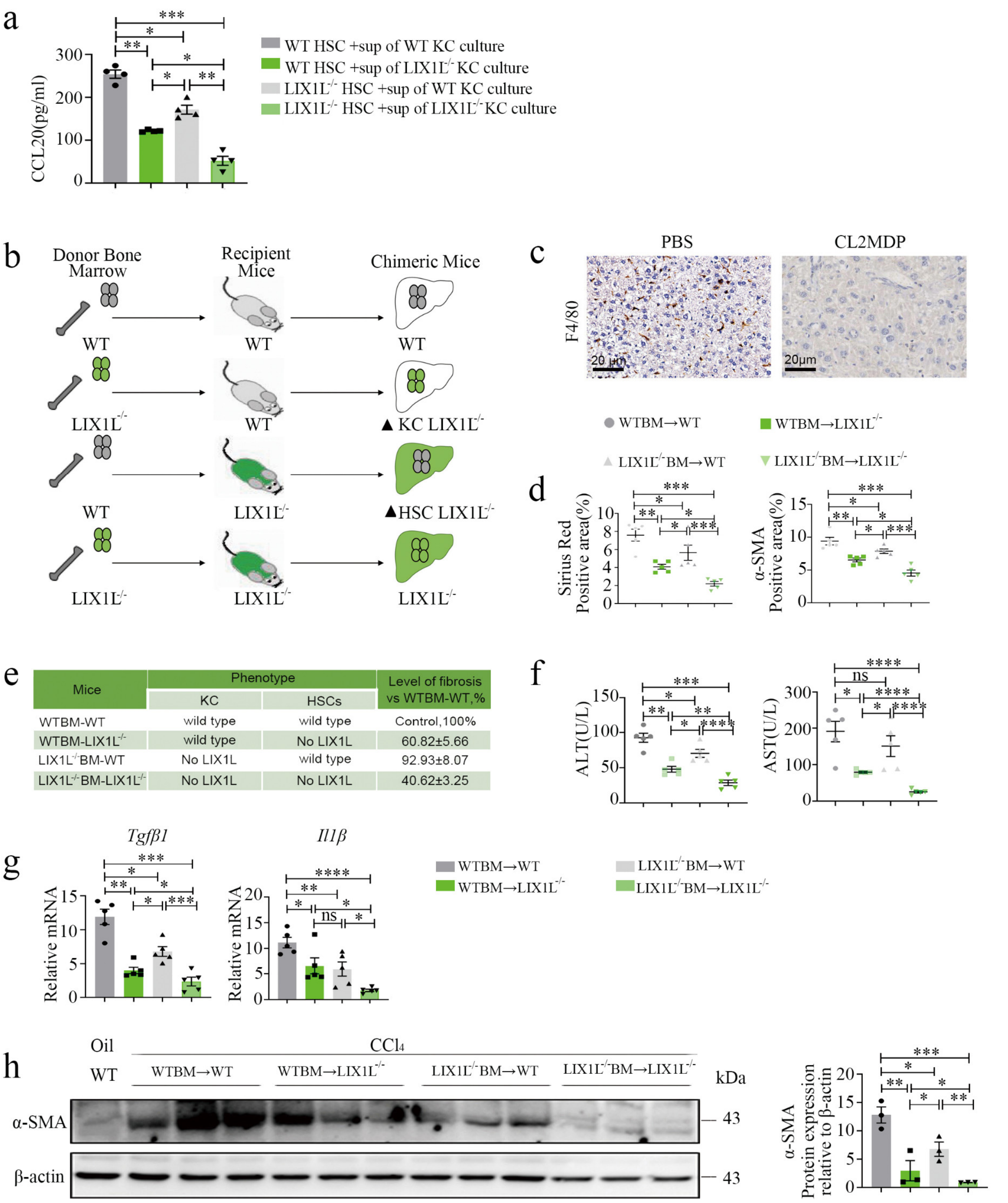

Supplementary Figure s8. (a) CCL20 secretion was measured in supernatant of WT and *Lix1l*$^{-/-}$ HSCs with WT or *Lix1l*$^{-/-}$ KC supernatant. (b) Illustration for production of chimeric mice by BMT (n=5 per group). (c) Representative images of F4/80 IHC staining. Mice were administered with CL2MDP (10 μl/g; i.p.) or PBS for 24 h followed by sacrifice. Livers were harvested and performed by IHC staining. (d) The positive area percent of Sirius Red and α-SMA. (e) The percent of liver fibrosis was calculated for BM chimeric mice in response to liver injury (in comparison WT BM→WT mice) based on Sirius Red and α-SMA expression. (f) The serum levels of ALT and AST. (g) Hepatic mRNA levels of *Tgfβ1* and *Il1β* in chimeric mice were measured by RT-Qpcr (n=5 per group)). (h) Hepatic expression of α-SMA in chimeric mice was measured by immunoblotting assay. Data are presented as means ± SEM. *P<0.05; **P<0.01;

***$P<0.001$; **** $P<0.0001$. NS: non-significant.
